# Tea intake and cardiovascular disease: an umbrella review

**DOI:** 10.1080/07853890.2021.1933164

**Published:** 2021-08-14

**Authors:** Abby Keller, Taylor C. Wallace

**Affiliations:** a Department of Nutrition and Food Studies, George Mason University, Fairfax, VA, USA; b Think Healthy Group, WA, USA

**Keywords:** Tea, cardiovascular diseases, Camellia sinensis, flavonoids, heart diseases

## Abstract

Brewed tea (*Camellia sinensis*) is a major dietary source of flavonoids, in particular flavan-3-ols. Tea consumption has been suggested to be inversely associated with a decreased risk of cardiovascular disease (CVD). Several biological mechanisms support the inverse relationship between tea flavonoid intake and CVD risk. Given the recent accumulating evidence from various systematic reviews regarding the role of tea as a beverage in reducing CVD risk and severity, we conducted an umbrella review to describe and critically evaluate the totality of evidence to date. We searched the PubMed, Web of Science, Cochrane Database of Systematic Reviews, and BIOSIS databases for systematic reviews published between January 1, 2010 and February 22, 2020 reporting relationships between tea (*C. sinensis*) consumption and CVD mortality, CVD diagnosis or incidence, CVD events, stroke events, blood pressure, endothelial function, blood lipids and triglycerides, and inflammatory markers. Herein, we describe results from 23 included systematic reviews. Consistently consuming 2 cups of unsweet tea per day offers the right levels of flavonoids to potentially decrease CVD risk and its progression. This is supported by the consistency between a recent high-quality systematic review and dose-response meta-analyses of population-based studies demonstrating beneficial effects of consumption on CVD mortality, CVD events and stroke events and medium- to high-quality systematic reviews of intervention studies that further elucidate potential benefits on both validated (i.e., SBP, DBP, total cholesterol, and LDL-cholesterol) and emerging risk biomarkers of CVD (TNF-ɑ and IL-6). On the basis of this umbrella review, the consumption of tea as a beverage did not seem to be harmful to health; therefore, the benefits of moderate consumption likely outweigh risk. Future large, clinical intervention studies will provide better mechanistic insight with the ability to confirm the outcome effects shown across observational studies. The review protocol was registered on PROSPERO (https://www.crd.york.ac.uk/PROSPERO/) as CRD42020218159.

**KEY MESSAGES**
- It is reasonable to judge that 2 cups of unsweet tea per day has the potential to decrease CVD risk and progression due to its flavonoid content.
- The primary side effects of tea documented in human studies are hepatotoxicity and gastrointestinal disturbances (i.e., vomiting and diarrhea) after high-dose supplemental intake.
- Additional clinical research is needed to fully elucidate the effects of tea flavonoids on markers of CVD, as many studies were under-powered to detect changes.

## Graphical Abstract

**Figure:**
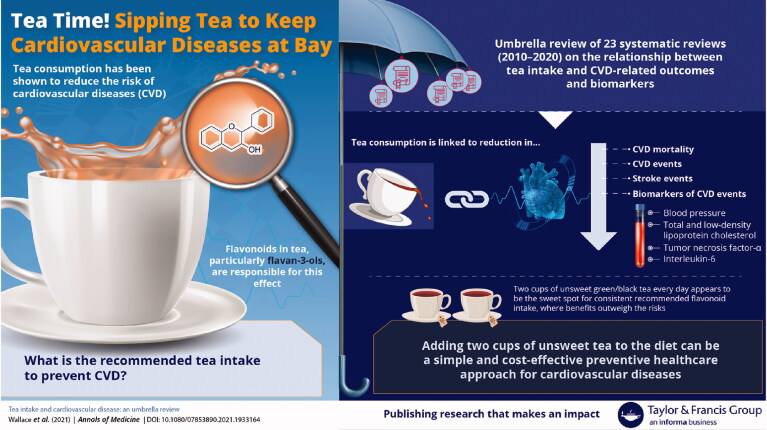

## Introduction

Brewed tea (*Camellia sinensis*) is a major dietary source of flavonoids, in particular flavan-3-ols [1]. Results of population studies commonly suggest that tea consumption is inversely associated with several health outcomes [2]. Shorter-term clinical intervention studies provide additional evidence that tea consumption has the potential to affect intermediate outcomes and biomarkers of disease in healthy, at-risk, and diseased populations [3,4]. Several recent systematic reviews have demonstrated these distinct effects, particularly in regard to cardiovascular disease (CVD) [2–4], the leading cause of death worldwide [5,6]. In the presence of suboptimal fruit, vegetable, and whole grain intake [7], unsweet tea may have increased potential to improve human health by providing consumers plant-derived dietary bioactive compounds such as flavonoids. This idea is supported by food intake data indicating that tea is a commonly consumed beverage worldwide; tea intake is secondary only to water [8]. Approximately one-fifth of Americans report drinking tea on a daily basis [9]. Tea consumers are shown to have about 20 times the flavonoid intake of non-consumers [9]. However, the amount of flavonoids and other dietary bioactives present in tea depends on several factors, including (1) their concentration in tea leaves; (2) the quantity of tea leaves used to prepare the infusion; (3) the volume of water used to prepare the infusion; (4) the water temperature, brew time, and agitation used to prepare the infusion; (5) the pH of the water used to prepare the infusion, thermal processing (for commercial preparations), and duration between preparation and consumption; and (6) the volume of tea consumed [1]. Similarly, the chemical composition of commercial tea extracts can vary greatly depending on the source material, extraction technique (e.g., water, alcohol, other solvent), type of industrial processing, and nature of the finished product.

Several biological mechanisms support the inverse relationship between tea flavonoid intake and CVD risk. The effects of tea flavonoids on endothelial function, nitric oxide–dependent vasorelaxation, and blood pressure are among the biological mechanisms cited most frequently in the peer-reviewed scientific literature [1,3,4,8,10,11]. The effects of tea on health are expected to be somewhat contingent on how tea is consumed (e.g., unsweet vs. sweetened). It is also important to note that exposures encountered from the use of pure compounds and tea extracts (e.g., dietary supplements) tend to be much higher in dose and have been shown to exhibit different efficacy and safety profiles. A 2018 systematic review of 159 intervention studies found that green tea preparations result in hepatic adverse events in a dose-dependent manner when ingested in large bolus amounts but not when consumed as brewed tea or extracts in beverages, or as a component of food [12]. According to the U.S. Department of Agriculture (USDA) Flavonoid Database [13], one cup (236.6 mL) of black or green tea provides approximately 280 or 338 mg of total flavonoids to the diet, respectively.

Given the recent accumulating evidence from various systematic reviews regarding the role of tea as a beverage in reducing CVD risk and severity, we conducted an umbrella review to describe and critically evaluate the totality of evidence to date.

## Materials and methods

An umbrella review of systematic reviews was conducted and is presented according to standardised methodology [14–16]. The review protocol was registered on PROSPERO (https://www.crd.york.ac.uk/PROSPERO/) as CRD42020218159. Two authors (TCW and AK) independently performed abstract and full-text screening, data extraction, and quality assessment of included systematic reviews. Discrepancies were resolved by consensus.

### Data sources and literature search

We searched the PubMed, Web of Science, Cochrane Database of Systematic Reviews, and BIOSIS databases for systematic reviews published between January 1, 2010 and February 22, 2020 reporting relationships between tea (*C. sinensis*) consumption and CVD mortality, CVD diagnosis or incidence, CVD events, stroke events, blood pressure, endothelial function, blood lipids and triglycerides, and inflammatory markers. Detailed search terms and strategies used for each database are presented in Supplemental Table 1. Missing data or additional information were requested from corresponding authors of articles when necessary.

**Table 1.** Characteristics of included systematic reviews of population-based studies.

Reference	Variable	Mean Age (y)	Study Population (Ag*e* ≥ 18 y)	Included Studies (N)	Follow-Up (y)	Quality (ROB) Assessment	Effects Model	Meta-Analysis Outcomes		Heterogeneity		Quality of Systematic Review
								RR (95% CI)	*p* Value	I^2^ (%)	*p* Value	
*CVD mortality*												
Chung et al. [2]	Green and black tea	NR	Healthy	17	5–18.7	New Castle-Ottawa	Mixed(dose-response)	0.96 (0.94 to 0.98)	.0001	72.4	.0001	High
	Green and black tea	<65	Healthy	15	7.7–18.7	New Castle-Ottawa	Mixed(dose-response)	0.98 (0.96 to 0.99)	.012	58.5	.002	
	Green and black tea	≥65	Healthy	4	5–11	New Castle-Ottawa	Mixed(dose-response)	0.89 (0.83 to 0.96)	.001	59.5	.06	
*CVD events* CVD events												
Chung et al. [2]	Green and black tea	NR	Healthy	7	5.6–15	New Castle-Ottawa	Mixed(dose-response)	0.98 (0.96 to 1.00)	.085	76.5	.0001	High
Zhang et al. [33]	Green and black tea	NR	NR	4	3.8–24	New Castle-Ottawa	Random (dose-response)	0.73 (0.53 to 0.99)	.045	NR	NR	Moderate
*Stroke events*												
Chung et al. [2]	Green and black tea	NR	Healthy	13	5–24	New Castle-Ottawa	Mixed(dose-response)	0.96 (0.93 to 0.99)	.002	63.9	.001	High
Shen et al. [29]	Green tea	20–89	Healthy	5	Median: 11.5	NR	Random (dose-response)	0.83 (0.72 to 0.96)	<.01	70.2	NR	Critically low
	Black tea	20–89	Healthy	13	Median: 11.5	NR	Random (dose-response)	0.91 (0.83 to 0.98)	0.17	26.8	NR	
Zhang et al. [33]	Green and black tea/placebo	NR	NR	2	3.8–24	New Castle-Ottawa	Random (dose-response)	0.82 (0.73 to 0.92)	.001	77	<.001	Moderate

95% CI = 95% confidence interval, NR = not reported, ROB = risk of bias, RR = relative risk.

### Study selection

Inclusion and exclusion criteria used to assess study eligibility incorporated the PICOS (Population, Intervention, Comparison, Outcome, Study design) format and are presented using the USDA Nutrition Evidence Systematic Review format [7]. Inclusion and exclusion criteria are presented in Supplemental Table 2. Systematic review eligibility was restricted to peer-reviewed, English-language studies in adults (aged ≥18 years). Systematic reviews that solely assessed effects of alcohol or solvent extracts of tea leaves, tea supplements, or herbal teas were excluded from this umbrella review. Reference lists of included systematic reviews were cross-checked to ensure no relevant systematic reviews were overlooked.

**Table 2.** Characteristics of included systematic reviews of clinical trials.

Reference	Intervention/ Control	Intervention/ Control (N)	Age (y)	Study Population (Ag*e* ≥ 18 y)	Included Studies (N)	Follow-Up	Quality (ROB) Assessment	Effects Model	Meta-Analysis Outcomes		Heterogeneity		Quality of Systematic Review
									Mean (95% CI)	*p* Value	I^2^ (%)	*p* Value	
*Total cholesterol*													
Araya-Quintanilla et al. [18]	Black tea/placebo	243/172	53.22	Hypercholesterolaemia or high cholesterol diagnosis	6	3–20 wk	Cochrane	Random	MD: −1.67 mg/dL (−5.47 to 8.80)	.65	100	<.00001	Low
Asbaghi et al. [19]	Green tea/placebo	300/212	50–65	Type2 diabetes	7	4–16 wk	Cochrane	Random	WMD: −6.81 mg/dL (−15.13 to 1.52)	.109	83	.000	Moderate
Hartley et al. [3]	Black tea/placebo	75/42	25–60	Healthy or high CVD risk	3	3–6 mo	Cochrane	Random	MD: NR	NR	84	NR	High
	Green tea/placebo	172/155	25–60	Healthy or high CVD risk	4	3–6 mo	Cochrane	Random	MD: −0.62 mmol/L (−0.77 t*o* − 0.46)	NR	N/A	NR	
Igho-Osagie et al. [49]	Green and black tea/placebo	184/136	≥18	Healthy	5	4–24 wk	Cochrane	Random	WMD: 6.82 mg/dL (−2.79 to 16.44	NR	0.0	.857	High
	Green and black tea/placebo	219/201	≥18	Atrisk of CVD	7	4–24 wk	Cochrane	Random	WMD: 1.36 mg/dL (−4.05 to 6.77	NR	0.0	.939	
Khalesi et al. [11]	Green tea/placebo	123/ 117	28.9–80	Healthy, or with hypertension, diabetes, or vascular disease	11	3–16 wk	Downs and Black’s	Random	MD: −0.15 mmol/L (−0.27 t*o* − 0.02)	NR	55	NR	Moderate
Kim et al. [22]	Green tea/placebo	NR	11–65	All (included some adolescents)	19	3–24 wk	ADA Research Design Implementation Checklist	Random	WMD: −5.46 mg/dL (−9.59 t*o* − 1.32)	NR	45	NR	Low
Li et al. [24]	Green and black tea/placebo	589/446	NR	Obese adults with BM*I* ≥ 25 kg/m^2^ and diagnosed with metabolic syndrome	12	NR	Cochrane	Random	SMD: −0.24 mmol/L (−0.47 to 0.00)	.05	68	NR	Moderate
	Black tea/placebo	138/446	NR	Obese adults with BM*I* ≥ 25 kg/m^2^ and diagnosed with metabolic syndrome	3	NR	Cochrane	Fixed	SMD: −0.16 mmol/L (−0.43 to 0.10)	.24	41	NR	
	Green tea/placebo	451/446	NR	Obese adults with BM*I* ≥ 25 kg/m^2^ and diagnosed with metabolic syndrome	9	NR	Cochrane	Random	SMD: −0.26 mmol/L (−0.57 to 0.05)	.10	74	NR	
Liu et al. [30]	Green and black tea/placebo	291/292	NR	Metabolic syndrome	10	NR	Cochrane	Random	SMD: −0.37 mmol/L (−1.01 to 0.26)	.290	93	<.01	Low
Onakpoya et al. [27]	Green tea/placebo	1487 (total)	6–71	Normotensive or hypertensive	19	3–24 wk	Independently assessed	Random	MD:−0.13 mmol/L (−0.2 to 0.07)	NR	8	<.0001	Moderate
Xu et al. [31]	Green tea/placebo	3024 (total)	NR	Normal weight, overweight, or obese	31	3 wk to 12 mo	Cochrane	Random	WMD:−4.66 mg/dL (−6.36 t*o* − 2.96)	<.0001	23.2	.124	Low
Zhao et al. [34]	Black tea/placebo	15–77	NR	Healthy, hypercholesterolaemia, prediabetes	10	3 wk to 6 mo	Jadad	Random	MD: −2.04 mg/dL (−6.43 to 2.35)	.363	0	.472	High
*LDL cholesterol*													
Araya-Quintanilla et al. [18]	Black tea/placebo	243/172	53	Hypercholesterolaemia	6	3–20 wk	Cochrane	Random	MD: −3.21 mg/dL (−11.02 to 4.60)	.42	100	<.0001	Low
Asbaghi et al. [19]	Green tea/placebo	277/192	50–65	Type2 diabetes	6	4–16 wk	Cochrane	Random	WMD: −0.37 mg/dL (−4.13 to 3.40)	.849	46.5	.082	Moderate
Hartley et al. [3]	Black tea/placebo	92/52	25–60	Healthy or high CVD risk	4	3–6 mo	Cochrane	Random	MD:−0.43 mmol/L (−0.56 t*o* − 0.31)	NR	34	<.0001	High
	Green tea/placebo	172/155	25–60	Healthy or high CVD risk	4	3–6 mo	Cochrane	Random	MD: −0.64 mmol/L (−0.77 t*o* − 0.52)	NR	21	<.0001	
Igho-Osagie et al. [49]	Green and black tea/placebo	149/114	≥18	Healthy	3	4–24 wk	Cochrane	Random	WMD: 3.84 mg/dL (−6.96 to 14.64	NR	0.0	.620	High
	Green and black tea/placebo	219/198	≥18	Atrisk of CVD	7	4–24 wk	Cochrane	Random	WMD: 0.72 mg/dL (−5.76 to 7.20	NR	19.4	.281	
Khalesi et al. [11]	Green tea/placebo	123/117	28.9–80	Healthy, or with hypertension, diabetes, or vascular disease	10	3–16 wk	Downs and Black’s	Random	MD: −0.16 mmol/L (−0.22 t*o* − 0.09)	NR	0	NR	Moderate
Kim et al. [22]	Green tea/placebo	NR	11–65	All (included some adolescents)	19	3–24 wk	ADA Research Design Implementation Checklist	Random	WMD: −5.30 mg/dL (−9.99 t*o* − 0.62)	NR	71	<.08	Low
Li et al. [24]	Green and black tea/placebo	589/446	NR	Obese adults with BM*I* ≥ 25 kg/m^2^ and diagnosed with metabolic syndrome	14	NR	Cochrane and Jadad	Random	SMD: −0.31 mmol/L (−0.55 t*o* − 0.06)	NR	75	.01	Moderate
	Black tea/placebo	589/446	NR	Obese adults with BM*I* ≥ 25 kg/m^2^ and diagnosed with metabolic syndrome	4	NR	Cochrane and Jadad	Random	SMD: −0.29 mmol/L (−1.02 to 0.43)	NR	89	.43	
	Green tea/placebo	589/446	NR	Obese adults with BM*I* ≥ 25 kg/m^2^ and diagnosed with metabolic syndrome	10	NR	Cochrane and Jadad	Random	SMD: −0.30 mmol/L (−0.52 t*o* − 0.08)	NR	59	.0008	
Liu et al. [30]	Green and black tea/placebo	305/306	NR	Metabolic syndrome	10	NR	Cochrane	Random	SMD: −0.63 mmol/L (−1.06 t*o* − 0.20)	.0057	NR	NR	Low
Momose et al. [26]	Green tea/placebo	1339 (total)	28–60	Healthy	17	3–14 wk	Jadad	Random	WMD: −7.38 mg/dL (−9.25 t*o* − 5.50)	<.00001	25	.16	Critically low
Onakpoya et al. [27]	Green tea/placebo	1422 (total)	6–71	Normotensive or hypertensive	17	3–24 wk	Independently assessed	Random	MD: −0.19 mmol/L (−0.3 to 0.09)	NR	70	.0004	Moderate
Xu et al. [31]	Green tea/placebo	3005 (total)	NR	Normal weight, overweight, or obese	29	3 wk to 12 mo	Cochrane	Random	WMD: −4.55 mg/dL (−6.31 t*o* − 2.80)	<.0001	28.1	.082	Low
Zhao et al. [34]	Black tea/placebo	15–77	NR	Healthy, hypercholesterolaemia, prediabetes	9	3 wk to 6 mo	Jadad	Random	MD: −4.64 mg/dL (−8.99 t*o* − 0.30)	.036	0	.584	High
*HDL cholesterol*													
Araya-Quintanilla et al. [18]	Black tea/placebo	243/172	53	Hypercholesterolaemia	6	3–20 wk	Cochrane	Random	MD: 0.38 mg/dL (−1.12 to 1.87)	.62	100	<.0001	Low
Asbaghi et al. [19]	Green tea/placebo	277/192	50–65	Type2 diabetes	6	4–16 wk	Cochrane	Random	WMD: −3.10 mg/dL (−10.16 to 3.95)	.389	95.4	.000	Moderate
Hartley et al. [3]	Black tea/placebo	90/56	25–60	Healthy or high CVD risk	4	3–6 mo	Cochrane	Random	MD: −0.01 mmol/L (−0.06 to 0.04)	NR	36	.02	High
	Green tea/placebo	177/155	25–60	Healthy or high CVD risk	4	3–6 mo	Cochrane	Random	MD: 0.01 mmol/L (−0.08 to 0.11)	NR	39	.18	
	Green tea/placebo	177/155	25–60	Healthy or high CVD risk	4	3–6 mo	Cochrane	Fixed	MD: −0.01 mmol/L (−0.27 to 0.07)	NR	NR	NR	
Igho-Osagie et al. [49]	Green and black tea/placebo	161/123	≥18	Healthy	4	4–24 wk	Cochrane	Random	WMD: −1.02 mg/dL (−5.65 to 3.61)	NR	19.1	.295	High
	Green and black tea/placebo	143/200	≥18	Atrisk of CVD	7	4–24 wk	Cochrane	Random	WMD: 1.16 mg/dL (−0.32 to 2.65)	NR	0.0	.829	
Khalesi et al. [11]	Green tea/placebo	123/117	28.9–80	Healthy, or with hypertension, diabetes, or vascular disease	9	3–16 wk	Downs and Black’s	Random	MD: 0.01 mmol/L (−0.05 to 0.06)	NR	49	.05	Moderate
Kim et al. [22]	Green tea/placebo	NR	11–65	All (included some adolescents)	19	3–24 wk	ADA Research Design Implementation Checklist	Random	WMD: −0.27 mg/dL (−1.26 to 1.09)	NR	51	>.6	Low
Li et al. [24]	Green and black tea/placebo	589/446	NR	Obese adults with BM*I* ≥ 25 kg/m^2^ and diagnosed with metabolic syndrome	9	NR	Cochrane and Jadad	Random	SMD: 0.18 mmol/L (0.01 to 0.35)	.03	52	.0004	Moderate
Liu et al. [30]	Green and black tea/placebo	305/306	NR	Metabolic syndrome	10	NR	Cochrane	Random	SMD: 0.13 mmol/L (−0.27 to 0.53)	.3629	NR	NR	Low
Momose et al. [26]	Green tea/placebo	1339 (total)	28–60	Healthy	17	3–14 wk	Jadad	Random	WMD:−0.07 mg/dL (−0.91 to 1.05)	.89	80	<.00001	Critically low
Onakpoya et al. [27]	Green tea/placebo	1344 (total)	6–71	Normotensive or hypertensive	17	3–24 wk	Independently assessed	Random	MD: −0.01 mmol/L (−0.08 to 0.06)	NR	90	.79	Moderate
Xu et al. [31]	Green tea/placebo	3073 (total)	NR	Normal weight, overweight/obese	29	3 wk to 12 mo	Cochrane	Random	WMD: 0.23 mg/dL (−0.45 to 0.91)	.50	34.8	.035	Low
Zhao et al. [34]	Black tea/placebo	15–77	NR	Healthy, hypercholesterolaemia, prediabetes	10	3 wk to 6 mo	Jadad	Random	MD: −1.15 mg/dL (−3.04 to 0.75)	.236	0	.616	High
*Triglycerides*													
Araya-Quintanilla et al. [18]	Black tea/placebo	243/172	53	Hypercholesterolaemia	6	3 wk to 20 wk	Cochrane	Random	MD: 0.28 mg/dL (−3.89 to 4.45)	.90	100	<.0001	Low
Asbaghi et al. [19]	Green tea/placebo	300/212	50–65	Type2 diabetes	7	4–16 wk	Cochrane	Random	WMD: −12.79 mg/dL (−24.74 t*o* − 0.84)	.036	69.8	.000	Moderate
Hartley et al. [3]	Black tea/placebo	93/56	25–60	Healthy or high CVD risk	4	3–6 mo	Cochrane	Random	MD: NR	NR	64	NR	High
	Green tea/placebo	172/155	25–60	Healthy or high CVD risk	4	3–6 mo	Cochrane	Random	MD: −0.08 mmol/L (−0.24 to 0.07)	.29	0	.41	
Igho-Osagie et al. [49]	Green and black tea/placebo	185/129	≥18	Healthy	5	4–24 wk	Cochrane	Random	WMD: 17.47 mg/dL (−1.40 to 36.34)	NR	0.0	.449	High
	Green and black tea/placebo	200/185	≥18	Atrisk of CVD	6	4–24 wk	Cochrane	Random	WMD: −4.80 mg/dL (−19.81 to 10.22)	NR	0.0	.531	
Khalesi et al. [11]	Green tea/placebo	123/117	28.9–80	Healthy, or with hypertension, diabetes, or vascular disease	10	3–16 wk	Downs and Black’s	Random	MD: 0.10 mmol/L (−0.13 to 0.32)	NR	80	<.0001	Moderate
Kim et al. [22]	Green tea/placebo	NR	11–65	All (included some adolescents)	19	3–24 wk	ADA Research Design Implementation Checklist	Random	WMD: −3.00 mg/dL (−2.73 to 8.73)	NR	0	>.6	Low
Li et al. [24]	Green and black tea/placebo	589/446	NR	Obese adults with BM*I* ≥ 25 kg/m^2^ and diagnosed with metabolic syndrome	13	NR	Cochrane and Jadad	Random	SMD: −0.13 mmol/L (−0.38 to 0.12)	.30	73	<.0001	Moderate
Liu et al. [30]	Green and black tea/placebo	245/246	NR	Metabolic syndrome	10	NR	Cochrane	Random	SMD: −0.03 mmol/L (−0.42 to 0.36)	.995	NR	NR	Low
Momose et al. [26]	Green tea/placebo	1339 (total)	28–60	Healthy	17	3–14 wk	Jadad	Random	WMD: −1.55 mg/dL (−7.32 to 10.41)	.73	95	<.00001	Critically low
Onakpoya et al. [27]	Green tea/placebo	1354 (total)	6–71	Normotensive or hypertensive	17	3–24 wk	Independently assessed	Random	MD: −0.02 mmol/L (−0.16 to 0.12)	NR	53	.79	Moderate
Xu et al. [31]	Green tea/placebo	3025 (total)	NR	Normal weight, overweight, or obese	29	3 wk to 12 mo	Cochrane	Random	WMD: 3.77 mg/dL (−8.90 to 1.37)	.15	56.5	.0001	Low
*Systolic blood pressure*													
Greyling et al. [20]	Black tea/placebo	378 (total)	33–73	Healthy, hypertensive	11	1–26 wk	Delphi	Random	MD: −1.8 mmHg (−2.80 t*o* − 0.70)	.0013	35	.11	Moderate
Hartley et al. [3]	Black tea/placebo	60/63	25–60	Healthy or high CVD risk	2	3–6 mo	Cochrane	Fixed	MD: −1.85 mmHg (−3.22 t*o* − 0.48)	.01	0	.49	High
	Green tea/placebo	83/84	25–60	Healthy or high CVD risk	2	3–6 mo	Cochrane	Fixed	MD:−3.18 mmHg (−5.25 t*o* − 1.11)	0	0	0.72	
Igho-Osagie et al. [49]	Green and black tea/placebo	104/104	≥18	Healthy	3	4–24 wk	Cochrane	Random	NR		NR	NR	High
	Green and black tea/placebo	127/126	≥18	Atrisk of CVD	5	4–24 wk	Cochrane	Random	WMD: 0.36 mg/dL (−3.04 to 3.75)	NR	28.7	.23	
Khalesi et al. [11]	Green tea/placebo	123/117	28.9–80	Healthy, or with hypertension, diabetes, or vascular disease	13	3–16 wk	Downs and Black’s	Random	MD: −2.08 mmHg (−3.06 t*o* − 1.05)	NR	0	NR	Moderate
Li et al. [23]	Green tea/placebo	971 (total)	29–54	Obese or overweight	14	3 wk to 3 mo	Cochrane	Random	MD: −1.42 mmHg (−2.47 t*o* − 0.36)	.008	52	.01	High
Li et al. [24]	Green and black tea/placebo	589/446	NR	Obese adults with BM*I* ≥ 25 kg/m^2^ and diagnosed with metabolic syndrome	9	NR	Cochrane and Jadad	Random	SMD: −0.16 mmHg (−0.41 to 0.09)	.20	64	.001	Moderate
Liu et al. [30]	Green and black tea/placebo	NR	NR	Metabolic syndrome	10	NR	Cochrane	Random	SMD: −0.83 mmHg (−1.75 to 0.09)	.0769	93	<.01	Low
Ma et al. [46]	Black tea/placebo	556/559	20–75	Healthy, elevated blood pressure, or hypertensive	12	1 day to 6 mo	Cochrane	Random	WMD:−1.04 mmHg (−2.05 t*o* − 0.03)	.04	71	.00001	Moderate
Mahdavi-Roshan et al. [25]	Green or black tea/placebo	203/205	52 ± 6	Hypertensive or elevated blood pressure	5	1–24 wk	Cochrane	Random	WMD: −4.81 mmHg (−8.4 t*o* − 1.58)	.004	93.9	<.001	Moderate
	Black tea/placebo	NR	52 ± 6	Hypertensive or elevated blood pressure	3	1–24 wk	Cochrane	Random	WMD: −2.67 mmHg (−6.37 t*o* − 1.04)	.158	78	.0003	
	Green tea/placebo	NR	52 ± 6	Hypertensive or elevated blood pressure	2	1–24 wk	Cochrane	Random	WMD: −6.22 mmHg (−9.92 t*o* − 2.52)	.001	91	.000	
Onakpoya et al. [27]	Green tea/placebo	1342 (total)	6–71	Normotensive or hypertensive	18	3–24 wk	Independently assessed	Random	MD:−1.94 mmHg (−2.95 t*o* − 0.93)	.0002	8	.36	Moderate
Yarmolisnky et al. [32]	Green or black tea/placebo	834 (total)	16–73	Prehypertensive/hypertensive	10	≥2 mo	Cochrane	Random	MD: −2.36 mmHg (−4.20 t*o* − 0.52)	NR	0	.99	Moderate
	Green or black tea/placebo	834 (total)	16–73	Prehypertensive/hypertensive	10	≥2 mo	Cochrane	Fixed	MD: −2.93 mmHg (−5.69 t*o* − 0.17)	NR	NR	NR	
*Diastolic blood pressure*													
Greyling et al. [20]	Black tea/placebo	378 (total)	33–73	Healthy or hypertensive	11	1–26 wk	Delphi	Random	MD: 1.3 mmHg (−1.80 t*o* − 0.80)	.0013	20	.26	Moderate
Hartley et al. [3]	Black tea/placebo	60/63	25–60	Healthy or high CVD risk	2	3–6 mo	Cochrane	Fixed	MD: −1.27 mmHg (−3.06 to 0.53)	NR	0	.53	High
	Green tea/placebo	83/84	25–60	Healthy or high CVD risk	2	3–6 mo	Cochrane	Fixed	MD: −3.42 mmHg (−4.54 t*o* − 2.30)	NR	0	.39	
Igho-Osagie et al. [49]	Green and black tea/placebo	104/104	≥18	Healthy	3	4–24 wk	Cochrane	Random	NR		NR	NR	High
	Green and black tea/placebo	127/126	≥18	Atrisk of CVD	5	4–24 wk	Cochrane	Random	WMD: 0.18 mmHg (−3.00 to 3.36)	NR	54.2	.068	
Khalesi et al. [11]	Green tea/placebo	123/117	28.9–80	Healthy, or with hypertension, diabetes, or vascular disease	13	3–16 wk	Downs and Black’s	Random	MD: −1.71 mmHg (−2.86 t*o* − 0.56)	NR	52	.02	Moderate
Li et al. [23]	Green tea/placebo	971 (total)	29–54	Obese or overweight	14	3 wk to 3 mo	Cochrane	Random	MD: −1.25 mmHg (−2.32 t*o* − 0.19)	.02	74	<.001	High
Li et al. [24]	Green and black tea/placebo	589/446	NR	Obese adults with BM*I* ≥ 25 kg/m^2^ and diagnosed with metabolic syndrome	9	NR	Cochrane and Jadad	Random	SMD: −0.16 mmHg (−0.47 to 0.16)	.20	77	<.00001	Moderate
Liu et al. [30]	Green and black tea/placebo	NR	NR	Metabolic syndrome	10	NR	Cochrane	Random	SMD: −0.89 mmHg (−1.73 to 0.05)	.0388	92	<.01	Low
Ma et al. [46]	Black tea/placebo	556/559	20–75	Healthy, elevated blood pressure, or hypertensive	12	1-day to 6 mo	Cochrane	Random	WMD:−0.59 mmHg (−1.05 t*o* − 0.13)	.01	71	.00001	Moderate
Mahdavi-Roshan et al. [25]	Green and black tea/placebo	203/205	52 ± 6	Hypertensive/elevated blood pressure	5	1–24 wk	Cochrane	Random	WMD: −1.98 mmHg (−3.77 t*o* − 0.20)	.029	86.3	<.001	High
	Black tea/placebo	NR	52 ± 6	Hypertensive/elevated blood pressure	3	1–24 wk	Cochrane	Random	WMD: −1.44 mmHg (−3.89 to 1.02)	.251	77	.004	
	Green tea/placebo	NR	52 ± 6	Hypertensive/elevated blood pressure	2	1–24 wk	Cochrane	Random	WMD: −2.36 mmHg (−4.8 to 0.09)	.059	85	.000	
Onakpoya et al. [27]	Green tea/placebo	1342 (total)	6–71	Normotensive or hypertensive	18	3–24 wk	Independently assessed	Random	MD: −0.98 mmHg (−2.14 to 0.18)	.1	62	NR	Moderate
Yarmolisnky et al. [32]	Green or black tea/placebo	834 (total)	16–73	Prehypertensive/hypertensive	10	≥2 mo	Cochrane	Random	MD: −1.77 mmHg (−3.03 t*o* − 0.52)	NR	0	.58	Moderate
	Green or black tea/placebo	834 (total)	16–73	Prehypertensive/hypertensive	10	≥2 mo	Cochrane	Fixed	MD: −2.40 mmHg (−4.22 t*o* − 0.57)	NR	NR	NR	
*Tumour necrosis factor-α*													
Haghighatdoost et al. [21]	Green tea/placebo	256 (total)	17–74.3	Free of acute inflammatory diseases	8	2–48 wk	Delphi	Random	WMD: −0.5 pg/mL (−0.96 t*o* − 0.03)	.036	96.9	.000	High
*C-reactive protein*													
Haghighatdoost et al. [21]	Green tea/placebo	887 (total)	17–74.3	Free of acute inflammatory diseases	15	2–48 wk	Delphi	Random	WMD: 0.05 mg/L (−0.18 to 0.28)	.692	96.8	.000	High
Li et al. [24]	Green and black tea/placebo	589/446	NR	Obese adults with BM*I* ≥ 25 kg/m^2^ and diagnosed with metabolic syndrome	6	NR	Cochrane and Jadad	Random	SMD: −0.21 mg/L (−0.77 to 0.34)	.45	85	NR	Moderate
	Black tea/placebo	589/446	NR	Obese adults with BM*I* ≥ 25 kg/m^2^ and diagnosed with metabolic syndrome	4	NR	Cochrane and Jadad	Random	WMD: −0.14 mg/L (−0.68 to 0.41)	.62	90	NR	
	Green tea/placebo	589/446	NR	Obese adults with BM*I* ≥ 25 kg/m^2^ and diagnosed with metabolic syndrome	2	NR	Cochrane and Jadad	Random	SMD: −0.10 mg/L (−0.10 to 0.91)	.85	85	NR	
Serban et al. [28]	Green tea/placebo	199/165	20–60	All adults	11	2 wk to 3 mo	Cochrane	Random	WMD: 0.085 mg/L (−0.225 to 0.395)	.592	NR	NR	Low
	Green tea/placebo	199/165	20–60	Healthy	11	2 wk to 3 mo	Cochrane	Random	WMD: −0.028 mg/L (−0.216 to 0.160)	.769	NR	NR	
	Green tea/placebo	199/165	20–60	Cardiometabolic diseases	11	2 wk to 3 mo	Cochrane	Random	WMD: 0.260 mg/L (−0.815 to 1.334)	.636	NR	NR	
*Interleukin-6*													
Haghighatdoost et al. [21]	Green tea/placebo	NR	17–74.3	Free of acute inflammatory diseases	7	2–48 wk	Delphi	Random	WMD: 1.38 pg/mL (0.13 to 2.63)	.031	96	<.0001	High
*Haemoglobin A1c*													
Li et al. [24]	Green and black tea/placebo	589/446	NR	Obese adults with BM*I* ≥ 25 kg/m^2^ and diagnosed with metabolic syndrome	3	NR	Cochrane and Jadad	Fixed	WMD: −0.14% (−0.39 to 0.10)	.26	0	NR	Moderate
	Black tea/placebo	589/446	NR	Obese adults with BM*I* ≥ 25 kg/m^2^ and diagnosed with metabolic syndrome	1	NR	Cochrane and Jadad	Fixed	WMD: −0.37% (−1.04 to 0.30)	.28	N/A	NR	
	Green tea/placebo	589/446	NR	Obese adults with BM*I* ≥ 25 kg/m^2^ and diagnosed with metabolic syndrome	1	NR	Cochrane and Jadad	Fixed	WMD: −0.11% (−0.38 to 0.16)	.43	0	NR	
Liu et al. [30]	Green and black tea/placebo	46/45	NR	Metabolic syndrome	10	NR	Cochrane	Random	SMD: 0.21% (−0.46 to 0.89)	.552	53	.14	Low
Xu et al. [50]	Green tea/placebo*	NR	NR	All adults	11	3 wk to 12 mo	Jadad	Random	WMD: −0.006% (−0.12 to 0.01)	.07	1.7	.43	Low

*Systematic review included studies absent of placebo (i.e. no intervention was the control arm).

ADA: American Dietetic Association; BMI: body mass index; CVD: cardiovascular disease; HDL: high-density lipoprotein; LDL: low-density lipoprotein; MD: mean difference; N/A: XX, NR: not reported; ROB: risk of bias; SMD: standard mean difference; WMD: weighted mean difference.

### Data extraction

Standardised data-extraction forms were used to abstract data from included systematic reviews of population-based and clinical studies separately. The following information was extracted independently from each eligible systematic review of population-based studies: the first author, year of publication, study population and age, number of included studies, follow-up time, outcomes, quality assessment tool(s), effects model(s), meta-analysis outcome(s), and heterogeneity. The following information was extracted independently from each eligible systematic review of clinical studies: the first author, year of publication, type of intervention and control, total number of individuals in the intervention and control, study population and age, number of included studies, follow-up time, outcomes, quality assessment tool(s), effects model(s), meta-analysis outcome(s), and heterogeneity. The estimated summary effect with its corresponding 95% confidence interval (95% CI) was extracted from meta-analyses of each included systematic review, when available, as was the I^2^ statistic.

### Evaluation of quality and grading of evidence

The methodological quality of all included systematic reviews was assessed using the AMSTAR (A MeaSurement Tool to Assess systematic Reviews) critical appraisal tool [17]. The AMSTAR 2 tool used in this review is an updated version of AMSTAR that enables more detailed assessment of systematic reviews that include randomised or nonrandomized studies of healthcare interventions. AMSTAR 2 has been shown to be a reliable and valid tool for assessing the quality of systematic reviews [17].

### Role of the funding source

No funding was accepted for this study.

## Results

**Figure 1.**
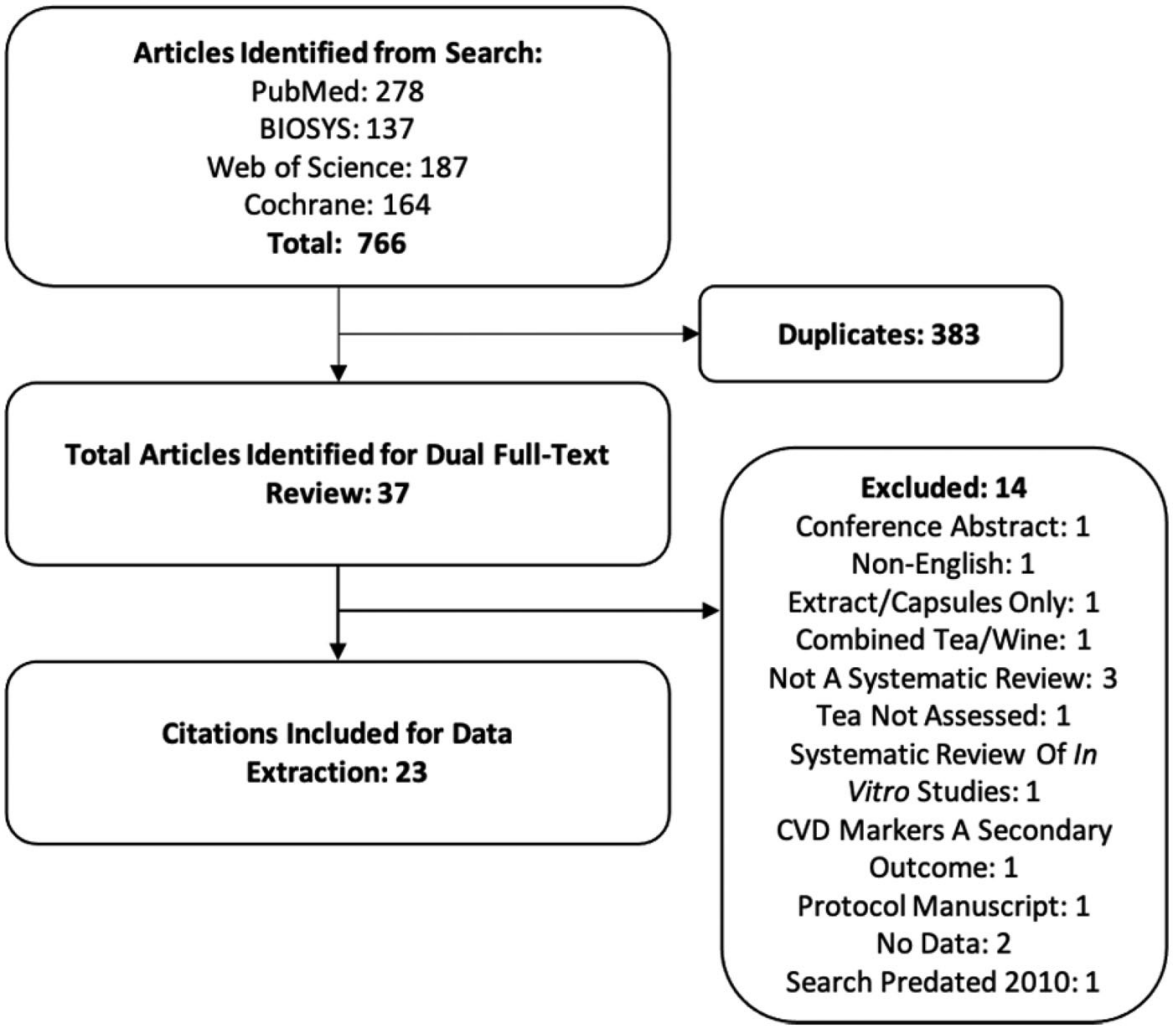
Flow-diagram of study selection process. CVD: cardiovascular disease.

Our literature search strategy identified 766 publications for dual title and abstract review. Of these, 383 were removed as duplicates. Thirty-seven publications were subject to dual full-text screening [2,3,11,18–51]. We excluded 14systematic reviews for the following reasons: one was a conference abstract [35], one was a non-English publication [44], one publication included only extracts or capsules [39], one study combined tea and wine with tea in the analyses [37], three were not systematic reviews [41,42,45], one publication did not assess tea intake [43], one publication was a systematic review of *in vitro* studies [36], one publication included CVD markers only as a secondary outcome [38], one publication was a protocol manuscript [51], two publications did not contain data [47,48], and one publication search predated 2010 [40]. Twenty-three publications [2,3,11,18–34,46] remained after dual full-text screening and searching the reference lists of included systematic reviews. The results of our systematic search and selection of eligible systematic reviews are shown in Figure 1. The quality of the systematic reviews varied from low to high. AMSTAR 2 strongly recommends that individual ratings not be combined to create an overall score and that the user should consider the potential impact of an inadequate rating for each item [17]. Lack of providing information on funding sources for studies included in each systematic review was the most common weakness detected in the included systematic reviews, followed by providing a list of excluded studies. The authors judged these two items to be noncritical weaknesses, and the quality assessments were rarely affected detrimentally by these items alone. The next common weakness among included systematic reviews was the absence of an explicit statement that the review methods were established prior to the conduct of the review. The authors judged this to be a critical weakness but made an attempt to search independently for registration in the Campbell Collaboration, Cochrane Collaboration, and PROSPERO databases when it was not reported explicitly within the study. Authors commonly reported that study selection or data extraction had been conducted induplicate, but not both. This may represent an error in reporting among systematic reviews and emphasises the need for peer-reviewed journals to better implement standards for reporting of systematic reviews.

### Systematic reviews of population-based studies

A total of three systematic reviews assessed effects of tea intake and CVD outcomes [2,29,33]. The results and AMSTAR2 quality assessment of these included systematic reviews are presented in Table 1.

#### CVD mortality

A single systematic review and mixed effects dose-response meta-analysis assessing effects of tea consumption on CVD mortality was identified from our literature search [2]. Meta-analysis of 19 studies showed that each cup (236.6 mL) increase in daily black or green tea was associated with a 4% lower risk of CVD mortality (pooled adjusted relative risk [RR]: 0.96; 95%CI: 0.94 to 0.98; *p* = .0001). Subgroup meta-analysis of four studies showed a greater magnitude of association in elderly individuals aged ≥65years; each cup (236.6 mL) increase in daily black or green tea was associated with a 11% lower risk of CVD mortality (pooled adjusted RR: 0.89; 95%CI: 0.83 to 0.96; *p* = .001) [2]. The quality of this systematic review was judged to be high (Table 1).

##### CVD Events

Two systematic reviews assessing effects of tea consumption on CVD events were identified from our literature search [2,33]. A recent systematic review and mixed effects dose-response meta-analysis of population-based studies showed tea consumption to have a significant inverse relationship with CVD events. Meta-analysis of seven studies showed that each cup increase in daily black or green tea was associated with a 2% lower risk of CVD events (pooled adjusted RR: 0.98; 95%CI: 0.96 to 1.00; *p* = .085) [2]. The quality of this systematic review was judged to be high. Consistent with this systematic review, a separate older systematic review and random effects dose-response meta-analysis of population-based studies showed tea consumption to have a significant relationship with CVD events. Meta-analysis of four studies showed that each 3-cup increase in daily black or green tea was associated with a 27% lower risk of CVD events (pooled adjusted RR: 0.73; 95%CI: 0.53to 0.99; *p* = .045) [33]. The quality of these systematic reviews was judged to be moderate to high (Table 1).

##### Stroke events

Three systematic reviews assessing effects of tea consumption on stoke events were identified from our literature search [2,29,33]. A recent systematic review and mixed effects dose-response meta-analysis of population-based studies showed tea consumption to have a significant inverse relationship with stroke events. Meta-analysis of 13 studies showed that each cup increase in daily black or green tea was associated with a 4% lower risk of stroke events (pooled adjusted RR: 0.96; 95%CI: 0.93 to 0.99; *p* = .002) [2]. The quality of this systematic review was judged to be high. Two older systematic reviews show consistent results. A former systematic review and random effects dose-response meta-analysis of population-based studies by Shen et al. showed tea consumption to have a significant relationship with stroke events. Meta-analysis of five studies showed that each 3-cup increase in daily black or green tea was associated with a 17% lower risk of CVDevents (pooled adjusted RR: 0.83; 95%CI: 0.72 to 0.96; *p* < .01) [29]. The quality of this systematic review was judged to be critically low. Another systematic review and random effects dose-response meta-analysis of population-based studies by Zhang et al. showed tea consumption to have a significant relationship with stroke events. Meta-analysis of two studies showed black or green tea to be associated with a 18% lower risk of CVD events (pooled adjusted RR: 0.82; 95% CI: 0.73 to 0.92; *p* = .001) [33]. The quality of this systematic review was judged to be moderate (Table 1).

### Systematic reviews of randomized controlled trials

#### Total cholesterol

Eleven systematic reviews assessing effects of tea consumption on total cholesterol were identified from our literature search [3,11,18,19,22,24,27,30,31,34,49]. Study populations ranged from healthy individuals and the general adult population to individuals with high CVD risk, hypercholesterolaemia, type-2 diabetes, prediabetes, obesity with metabolic syndrome, or hypertension. One study included some adolescents [22]. Li et al. found a significant decrease in total cholesterol among obese individuals with a body mass index (BMI) ≥25 kg/m^2^ diagnosed with metabolic syndrome who consumed green and black tea (combined) vs. placebo (standard mean difference [SMD]: 0.24 mmol/L; 95% CI: −0.47 to 0.00; *p* = .05) in a random effects meta-analysis [24]. No effects were reported for green tea vs. placebo or black tea vs. placebo [24]. Xu et al. found a significant decrease in total cholesterol among normal weight, overweight, and obese individuals (combined) who consumed green tea vs. placebo (weighted mean difference [WMD]: −4.66 mg/dL; 95% CI: 6.36 to −2.96; *p* = .0001) in a random effects meta-analysis [31]. The remaining nine older systematic reviews found no significant effects on total cholesterol across various populations [3,11,18,19,22,27,30,34,49], which may be due to a smaller number of studies and overall sample size. The quality of these systematic reviews was judged to be low to high (Table 2).

#### Low-density lipoprotein cholesterol

Twelve systematic reviews assessing effects of tea consumption on low-density lipoprotein (LDL) cholesterol were identified from our literature search [3,11,18,19,22,24,26,27,30,31,34,49]. Study populations ranged from healthy individuals and the general adult population to individuals with high CVD risk, hypercholesterolaemia, type-2 diabetes, prediabetes, obesity with metabolic syndrome, or hypertension. One study included some adolescents [22]. Li et al. found a significant decrease in LDL cholesterol among obese individuals (BMI ≥25 kg/m^2^) diagnosed with metabolic syndrome who consumed green and black tea (combined) vs. placebo (SMD: −0.31 mmol/L; 95% CI: −0.55 to −0.06; p = not reported [NR]) and green tea vs. placebo (SMD: −0.30 mmol/L; 95% CI: −0.52 to −0.08; p = NR) in a random effects meta-analysis [24]. No effects were reported for black tea vs. placebo [24]. Liu et al. found a significant decrease in LDL cholesterol among individuals with metabolic syndrome who consumed green and black tea (combined) vs. placebo in a random effects meta-analysis (SMD: −0.63 mmol/L; 95% CI: −1.06 to −0.20; *p* = .0057) [30]. Xu et al. found a significant decrease in LDL cholesterol among normal weight, overweight, and obese individuals (combined) who consumed green tea vs. placebo (WMD: −4.55 mg/dL; 95% CI: −6.31 to −2.80 mg/dL; *p* = .0001) in a random effects meta-analysis [31]. Momose et al. found a significant decrease in LDL cholesterol among healthy individuals who consumed green tea vs. placebo (WMD: −7.38 mg/dL; 95% CI: −9.25 to −5.50; *p* < .00001) in a random effects meta-analysis [26]. Zhao et al. found a significant decrease in LDL cholesterol among healthy, hypercholesterolemic, and prediabetic individuals who consumed black tea vs. placebo (mean difference [MD]: −4.64 mg/dL; 95% CI: −8.99 to −0.30; *p* = .036) in a random effects meta-analysis [34]. Khalesi et al. found a significant decrease in LDL cholesterol among healthy, hypertensive, and diabetic individuals, and those with vascular disease who consumed green tea vs. placebo (MD: −0.16 mmol/L; 95% CI: −0.22 to −0.09; *p* = NR) in a random effects meta-analysis [11]. Hartley et al. conducted a Cochrane review and found a significant decrease in LDL cholesterol among healthy and high-risk individuals who consumed black tea vs. placebo (MD: −0.43 mmol/L; 95% CI: −0.56 to −0.31; *p* = NR) and green tea vs. placebo (MD: −0.64 mmol/L; 95% CI: −0.77 to −0.52; *p* = NR) in a random effects meta-analysis [3]. Kim et al. found a significant decrease in LDL cholesterol among individuals of various health status who consumed green tea vs. placebo (WMD: −5.30 mg/dL; 95% CI: −9.99 to −0.62; *p* = NR) in a random effects meta-analysis [22]. The remaining four older systematic reviews found no significant effects on LDL cholesterol across various populations [18,19,27,49], which may be due to a smaller number of studies and overall sample size. The quality of these systematic reviews was judged to be critically low to high (Table 2).

#### High-density lipoprotein cholesterol

Twelve systematic reviews assessing effects of tea consumption on high-density lipoprotein (HDL) cholesterol were identified from our literature search [3,11,18,19,22,24,26,27,30,31,34,49]. Study populations ranged from healthy individuals and the general adult population to individuals with high CVD risk, hypercholesterolaemia, type-2 diabetes, prediabetes, obesity with metabolic syndrome, or hypertension. One study included some adolescents [22]. These systematic reviews consistently failed to show significant effects on HDL cholesterol across various populations [3,11,18,19,22,24,26,27,30,31,34,49]. The quality of these systematic reviews was judged to be critically low to high (Table 2).

#### Triglycerides

Eleven systematic reviews assessing effects of tea consumption on triglycerides were identified from our literature search [3,11,18,19,22,24,26,27,30,31,49]. Study populations ranged from healthy individuals and the general adult population to individuals with high CVD risk, hypercholesterolaemia, type 2 diabetes, prediabetes, obesity with metabolic syndrome, or hypertension. One study included some adolescents [22]. Asbaghi et al. (2020) found a significant decrease in triglycerides among individuals with type 2 diabetes who consumed green tea vs. placebo (WMD: −12.79 mg/dL; 95% CI: −24.74 to −0.84; *p* = .036) in a random effects meta-analysis [19]. The remaining 10 systematic reviews found no significant effects on triglycerides across various populations [3,11,18,22,24,26,27,30,31,49]. The quality of these systematic reviews was judged to be critically low to high (Table 2). Inconsistency may be due to differences in health status of participants. The systematic reviews also had an overall small sample size and differences in the how participants (e.g. healthy, at-risk, and diseased) were grouped in the analyses.

#### Systolic blood pressure

Eleven systematic reviews assessing effects of tea consumption on systolic blood pressure (SBP) were identified from our literature search [3,11,20,23–25,27,30,32,46,49]. Study populations ranged from healthy individuals and the general adult population to individuals with high CVD risk, hypercholesterolaemia, type-2 diabetes, prediabetes, obesity with metabolic syndrome, or hypertension. Ma et al. found a significant decrease in SBP among individuals with hypertension or elevated blood pressure who consumed black tea vs. placebo (WMD: −1.04 mmHg; 95% CI: −2.05 to −0.03; *p* = .04) [46]. Mahdavi-Roshan et al. (2020) found a significant decrease in SBP among individuals with hypertension or individuals with elevated blood pressure who consumed green or black tea vs. placebo (WMD: −4.81 mmHg; 95% CI: −8.4 to −1.58; *p* = .004), black tea vs. placebo (WMD: −2.67 mmHg; 95% CI: −6.37 to −1.04; *p* = .158), and green tea vs. placebo (WMD: −6.22 mmHg; 95% CI: −9.92 to −2.52; *p* = .001) in a random effects meta-analysis [25]. Li et al. found a significant decrease in SBP among obese or overweight individuals who consumed green tea vs. placebo (MD: −1.42 mmHg; 95% CI: −2.47 to −0.36; *p* = .008) in a random effects meta-analysis [23]. Yarmolisnky et al. found a significant decrease in SBP among prehypertensive and hypertensive individuals who consumed green or black tea vs. placebo in a random effects (MD: −2.36 mmHg; 95% CI: −4.20 to −0.52; *p* = NR) and fixed effects meta-analysis (MD: −2.93 mmHg; 95% CI: −5.69 to −0.17; *p* = NR) [32]. Greyling et al. (2014) found a significant decrease in SBP among healthy and hypertensive individuals who consumed green or black tea vs. placebo (MD: −1.8 mmHg; 95% CI: −2.80 to −0.70; *p* = .0013) in a random effects meta-analysis [20]. Khalesi et al. found a significant decrease in SBP among healthy individuals and those with hypertension, diabetes, and vascular disease who consumed green tea vs. placebo (MD: −2.08 mmHg; 95% CI: −3.06 to −1.05; *p* = NR) in a random effects meta-analysis [11]. Onakpoya et al. found a significant decrease in SBP among normotensive or hypertensive individuals who consumed green tea vs. placebo (MD: −1.94 mmHg; 95% CI: −2.95 to −0.93; *p* = NR) in a random effects meta-analysis [27]. Hartley et al. conducted a Cochrane review and found a significant decrease in SBP among healthy and high-risk individuals who consumed black tea vs. placebo in (MD: −1.85 mmHg; 95% CI: −3.22 to −0.48; *p* = .01) and green tea vs. placebo (MD: −3.18 mmHg; 95% CI: −5.25 to −1.11; *p* = .00) in a fixed effects meta-analysis [3]. The remaining three older systematic reviews found no significant effects on SBP across various populations [24,30,49], which may be due to a smaller number of studies and overall sample size. The quality of these systematic reviews was judged to be low to high (Table 2).

#### Diastolic blood pressure

Eleven systematic reviews assessing effects of tea consumption on diastolic blood pressure (DBP) were identified from our literature search [3,11,20,23–25,27,30,32,46,49]. Study populations ranged from healthy individuals and the general adult population to individuals with high CVD risk, hypercholesterolaemia, type-2 diabetes, prediabetes, obesity with metabolic syndrome, or hypertension. Ma et al. found a significant decrease in DBP among individuals with hypertension or elevated blood pressure who consumed black tea vs. placebo (WMD: −0.59 mmHg; 95% CI: −1.05 to −0.13; *p* = .01) [46]. Mahdavi-Roshan et al. (2020) found a significant decrease in DBP among individuals with hypertension or elevated blood pressure who consumed green or black tea vs. placebo (WMD: −1.98 mmHg; 95% CI: −3.77 to − 0.20; *p* = .029), but not black tea vs. placebo or green tea vs. placebo, in a random effects meta-analysis [25]. Li et al. found a significant decrease in DBP among obese or overweight individuals who consumed green tea vs. placebo (MD: −1.25 mmHg; 95% CI: −2.32 to −0.19; *p* = .02) in a random effects meta-analysis [23]. Yarmolisnky et al. (2015) found a significant decrease in DBP among prehypertensive and hypertensive individuals who consumed green or black tea vs. placebo in a random effects (MD: −1.77 mmHg; 95% CI: −3.03 to −0.52; *p* = NR) and fixed effects meta-analysis (MD: −2.40 mmHg; 95% CI: −4.22 to −0.57; *p* = NR) [32]. Greyling et al. found a significant decrease in DBP among healthy and hypertensive individuals who consumed green or black tea vs. placebo (MD: −1.3 mmHg; 95% CI: −1.80 to −0.80; *p* = .0013) in a random effects meta-analysis [20]. Khalesi et al. found a significant decrease in DBP among healthy individuals and in those with hypertension, diabetes, and vascular disease who consumed green tea vs. placebo (MD: −1.71 mmHg; 95% CI: −2.86 to −0.56; *p* = NR) in a random effects meta-analysis [11]. Hartley et al. conducted a Cochrane review and found a significant decrease in DBP among healthy and high-risk individuals who consumed green tea vs. placebo (MD: −3.42 mmHg; 95% CI: −4.54 to −2.30; *p* = NR), but not black tea vs. placebo, in a fixed effects meta-analysis [3]. The remaining three older systematic reviews found no significant effects on DBP across various populations [24,27,30,49] which may be due to a smaller number of studies and overall sample size.The quality of these systematic reviews was judged to be low to high (Table 2).

#### Tumour necrosis factor-α

One systematic review assessing effects of tea consumption on tumour necrosis factor-α (TNF-α) was identified from our literature search [21]. Haghighatdoost et al. found a significant decrease in TNF-α among individuals free of acute inflammatory diseases who consumed green tea vs. placebo (WMD: −0.5 pg/mL; 95% CI: −0.96 to −0.03; *p* = .036) [21]. The quality of this systematic review was judged to be high (Table 2).

#### C-reactive protein

Three systematic reviews assessing effects of tea consumption on C-reactive protein (CRP) were identified from our literature search [21,24,28]. Study populations ranged from healthy individuals to those with obesity with metabolic syndrome or cardiometabolic diseases. None of the three systematic reviews found significant effects on CRP across various populations. The quality of these systematic reviews was judged to be low to high (Table 2).

#### Interleukin-6

One systematic review assessing effects of tea consumption on interleukin-6 (IL-6) was identified from our literature search [21]. Haghighatdoost et al. found a significant increase in IL-6 among individuals free of acute inflammatory diseases who consumed green tea vs. placebo (WMD: −1.38 pg/mL; 95% CI: 0.13 to 2.63; *p* = .031) [21]. The quality of this systematic review was judged to be high (Table 2).

#### Haemoglobin A1c

Three systematic reviews assessing effects of tea consumption on haemoglobin A1c (HbA1c) were identified from our literature search [24,30,50]. Study populations ranged from individualswith metabolic syndrome to those who were obese with metabolic syndrome. None of the systematic reviews found significant effects on HbA1c across various populations [24,30,50]. The quality of these systematic reviews was judged to be low to moderate (Table 2).

## Discussion

In this umbrella review, we describe results from 23 systematic reviews that investigated the role of tea in reducing CVD risk. Based on the scientific literature presented, it is reasonable to judge that 2cups of unsweet tea per day has the potential to decrease CVD risk and progression due to its flavonoid content. Aside from the included systematic reviews, it is important to note that a recent two-sample Mendelian randomisation analysis provided evidence that a genetically predicted extra daily cup of tea is associated with a reduced risk of small vessel stroke (odds ratio: 0.79; 95% CI: 0.69 to 0.91; *p* = .0001) [52], lending greater biological plausibility to our findings. Tea flavonoids seem to decrease LDL cholesterol, SBP, and DBP across both healthy and at-risk populations, although larger study sample sizes and consistency limit these findings. Beneficial effects seem to be most distinct when healthy and at-risk populations are analysed together, alluding to the sample size issues noted above and by Igho-Osagie et al. [49]. It is possible that the sample sizes present in most intervention studies were insufficient to show modest effects on biomarkers such as blood pressure and lipids. Differences in tea consumed as a beverage vs. supplement also seem to exist, as demonstrated when comparing the systematic reviews from Igho-Osagie et al. [49] and Hartley et al. [3]. Igho-Osagie et al. [49] did not include studies in which tea extracts or supplements were administered as the intervention. Adherence issues are likely to be more apparent across whole food or beverage versus supplemental interventions. Adding to this conundrum, the systematic reviews reviewed in this umbrella review failed to provide sufficient information on the variation in chemical composition of tea beverages, supplements, and/or extracts used among included intervention studies.

Other mechanisms may also be responsible for the effects seen on CVD incidence, and outcomes seen across observational studies. Tea flavonoids have been thought to improve blood flow by enhancing endothelial nitric oxide bioavailability [4]. During the past two decades, flow-mediated dilation (FMD) has been used increasingly as a tool to assess effects of interventions on endothelial function in humans. Several (but not all) prospective studies indicate inverse relationships between FMD and CVD risk in healthy and diseased individuals [10–12,14–19]. An earlier systematic review of randomised controlled trials predating our literature search found that a mean daily dose of 500 mL of tea (about 2cups) increased FMD by about 2.6% arterial diameter (95% CI: 1.9to3.3%; *p* < .001; *n* = 9) compared to placebo [4]. An updated systematic review in this area is needed. Tea flavonoids seem to exert beneficial effects on some but not all markers of inflammation; however, larger controlled intervention studies are needed to fully elucidate these effects.

Since tea consumption is widespread and associated with multiple health attributes, it is also important to consider potential toxicological effects. Nervousness, anxiety, heart irregularities, headaches, tremors, hypotension, restlessness, insomnia, irritation of the gastrointestinal mucosa, diuresis, and daytime irritability are some potential side effects of tea consumption [53,54]. Most of these effects were shown when tea was consumed during a fasted state. The primary side effects documented in human studies are hepatotoxicity and gastrointestinal disturbances (i.e. vomiting and diarrhoea) after high-dose supplemental intake [54]. A 2018 systematic review assessed adverse events among 159 human intervention studies and found catechin-rich green tea preparations to result in hepatic adverse events in a dose-dependent manner when ingested in large amounts *via* supplements but not when consumed as brewed tea, extracts in beverages, or as part of food [12]. Toxicological evidence from laboratory studies suggests that hepatotoxic effects are strongly associated with certain dosing conditions (e.g. bolus dose *via* gavage, fasting) and positively correlated with total catechin and epigallocatechin gallate (EGCG) content. This systematic review concluded that an observed safe level of 704 mg of EGCG per day might be considered for green tea preparations in beverage form based on human adverse event data [12]. According to the USDA Flavonoid Database [13], brewed green tea contains an average of 126.6 mg of total catechins and 77.8 mg of EGCG per 100 mL as consumed, on the basis of 1 g of leaf/100 mL of infusion. It should be noted that the larger amount of flavonoids present in tea extracts or supplements may exhibit a variety of drug–nutrient interactions that are likely not apparent through beverage tea intake [55]. There are limited data in pregnant and lactating women and among other susceptible subpopulations. Consumption of tea as a beverage in humans at physiologically reasonable levels appears to be safe.

Our umbrella review has several strengths and limitations. Quality assessment of the included systematic reviews by AMSTAR 2 is a major strength. Our umbrella review is limited due to the reliance on previously published systematic reviews, leaving the opportunity that missed studies may have an influence on our findings. To circumvent this dilemma, we included all (not just the most recent) systematic reviews within the past 10 years and made judgements based on the consistency of evidence over time. There is known overlap between studies included amongst similar systematic reviews; however, consistency of the results reported by different researchers analysing similar data reduces the chance of confirmation bias. As previously stated, the small sample sizes present in many meta-analyses within included systematic reviews may also be a limiting factor for detecting more modest effects of tea. Additional large, randomised intervention studies of both healthy and at-risk populations assessing the effects of beverage tea intake on CVD outcomes and biomarkers can help increase the evidence supporting our reported effects.

## Conclusion

A consistent intake of tea at levels around 2cups per day seems to have the potential to decrease CVD risk and progression due to its flavonoid content. This is supported by the consistency between a recent high-quality systematic review and dose-response meta-analyses of population-based studies demonstrating beneficial effects of consumption on CVD mortality, CVD events and stroke events and medium- to high-quality systematic reviews of intervention studies that further elucidate potential benefits on both validated (i.e., SBP, DBP, total cholesterol, and LDL-cholesterol) and emerging risk biomarkers of CVD (TNF-ɑ and IL-6). On the basis of this umbrella review, the consumption of tea as a beverage did not seem to be harmful to health; therefore, the benefits of moderate consumption likely outweigh risk. Future large, clinical intervention studies are needed to provide additional mechanistic insight (e.g., effects on FMD) to confirm the outcome effects shown across observational studies.
